# One of the Objects of the Register

**Published:** 1848-07

**Authors:** 


### ONE OF THE OBJECTS OF THE REGISTER.

There are some members of the Profession very far in advance of their fellows. Acquaint them with any new, or supposed new improvement—why, they have thus practiced for years, or knew of it, or had a much superior plan. Now, there are many things thus locked up that would be very useful to the profession. Indeed all the improvements to be made in the next quarter of a century are now known by these wiseacres, but we very much fear that they will lose the credit due to discoverers by interlopers dropping in and picking up scraps, and then proclaiming themselves the true discoverers, when in fact it had been known, long known by others, who supposed that because they knew it all must have known it. We would, however, set it down as a rule, that when any one finds himself enjoying a mode or facility of practice not mentioned in any of the publications, that they consider it new, and send an account of it to the Register, one of the objects of which is to make useful knowledge common to all; and if they do not embrace the opportunity thus offered to secure to themselves their own honors, we shall be very much inclined to set them down as not knowing so much more than the rest of us, after all.

